# Management of Adverse Events Associated with Pomalidomide-Based Combinations in Patients with Relapsed/Refractory Multiple Myeloma

**DOI:** 10.3390/cancers16051023

**Published:** 2024-02-29

**Authors:** Omar Nadeem, Sikander Ailawadhi, Jack Khouri, Louis Williams, Donna Catamero, Kathryn Maples, Jesús Berdeja

**Affiliations:** 1 Dana-Farber Cancer Institute, Harvard Medical School, Boston, MA 02215, USA; 2 Department of Hematology & Oncology, Mayo Clinic, Jacksonville, FL 32224, USA; ailawadhi.sikander@mayo.edu; 3 Department of Hematology and Medical Oncology, Cleveland Clinic, Cleveland, OH 44195, USA; khourij@ccf.org (J.K.); willial47@ccf.org (L.W.); 4 Mount Sinai Hospital, New York, NY 10029, USA; donna.catamero@mountsinai.org; 5 Department of Pharmacy, Winship Cancer Institute, Emory University, Atlanta, GA 30322, USA; kathryn.maples@gmail.com; 6 Greco-Hainsworth Centers for Cancer Research, Tennessee Oncology, Nashville, TN 37203, USA; jberdeja@tnonc.com

**Keywords:** relapsed/refractory multiple myeloma, immunomodulatory (IMiD^®^) agents, pomalidomide, combination therapy, adverse events, safety, treatment selection, management, case studies, cereblon E3 ligase modulator (CELMoD™) agents

## Abstract

**Simple Summary:**

Pomalidomide is one of three immunomodulatory (IMiD^®^) agents approved for the treatment of multiple myeloma. Despite providing survival benefits, IMiD agents have distinct but overlapping safety profiles. These safety profiles should be considered at the treatment selection stage and when monitoring and managing adverse events to help patients adhere to their treatments, therefore maximizing their effectiveness and helping to maintain quality of life. Here, we discuss common adverse events associated with pomalidomide and present five clinically relevant hypothetical case studies in patients with relapsed/refractory multiple myeloma. We discuss factors impacting treatment selection and provide guidance on how to mitigate and manage adverse events based on published evidence and clinical experience. We also offer future perspectives by briefly discussing targeted protein degraders, a new class of medications currently being studied in clinical trials for the treatment of myeloma that build upon the well-established IMiD agent platform.

**Abstract:**

Multi-agent regimens incorporating immunomodulatory (IMiD^®^) agents such as thalidomide, lenalidomide, and pomalidomide have become the preferred standard of care for the treatment of patients with multiple myeloma (MM), resulting in improved survival outcomes. Currently, there are three IMiD agents approved for the treatment of MM: thalidomide, lenalidomide, and pomalidomide. Lenalidomide is commonly used to treat patients with newly diagnosed MM and as maintenance therapy following stem cell transplant or after disease relapse. Pomalidomide, the focus of this review, is approved in patients with relapsed/refractory MM (RRMM). Despite survival benefits, IMiD agents each have different safety profiles requiring consideration both prior to starting therapy and during treatment. Adverse event (AE) management is essential, not only to ensure treatment adherence and thus ensure optimal efficacy but also to maintain patient quality of life. Here, we discuss AEs associated with pomalidomide and present five clinically relevant hypothetical case studies in patients with RRMM to provide scenario-driven guidance regarding treatment selection and AE prevention and management in the clinical setting. Lastly, as new treatment approaches continue to be explored in MM, we also discuss novel cereblon E3 ligase modulator (CELMoD™) agents including iberdomide (CC-220) and mezigdomide (CC-92480).

## 1. Introduction

Immunomodulatory (IMiD^®^) agents including thalidomide, lenalidomide, and pomalidomide have become part of the backbone for the treatment of patients with multiple myeloma (MM), leading to improved response and survival rates [1,2,3,4]. In MM, treatment with an IMiD agent forms part of the preferred standard of care as a component of triplet and quadruplet regimens, with agents such as pomalidomide increasingly being used [1]. This has resulted in improvements in survival while also benefiting patient quality of life (QOL) [5,6,7,8,9]. Although these agents are associated with relatively lower toxicity than chemotherapy, treatment with IMiD agents nonetheless results in a range of adverse events (AEs) that require special attention and management to enable patients to adhere to their treatments. Although thalidomide, lenalidomide, and pomalidomide are structurally similar, they are associated with differing safety profiles (Table 1). Thalidomide is particularly associated with sedation, constipation, peripheral neuropathy, and muscle weakness [1,10]. Lenalidomide is associated with an increased risk of second primary malignancies (SPMs; particularly hematological malignancies), rash, diarrhea, constipation, hematological toxicities such as neutropenia and thrombocytopenia, myocardial infarction, venous thromboembolism (VTE), arterial thromboembolism, and stroke [3,11,12,13]. Of note, the risk of thromboembolism leading to heart attacks or stroke is increased when lenalidomide is combined with dexamethasone [3]. There is also evidence that lenalidomide promotes the development of TP53-mutated myeloid neoplasms [14].

Pomalidomide, an IMiD agent and the focus of this review, was approved in 2013 for the treatment of patients with relapsed and/or refractory MM (RRMM) who had received ≥2 prior regimens including lenalidomide and bortezomib [4]. Pomalidomide is associated with a lower incidence of gastrointestinal side effects and is less dependent on renal function than thalidomide and lenalidomide. As with lenalidomide, pomalidomide is associated with an increased risk of VTE, myocardial infarction, and stroke (for which antithrombotic prophylaxis is recommended in patients with known risk factors), especially when combined with dexamethasone [4]. In patients treated with pomalidomide monotherapy, the hematologic toxicities neutropenia, anemia, and thrombocytopenia occur in approximately 53%, 38%, and 26% of patients, respectively; grade 3/4 events occur in approximately 48%, 23%, and 22% [2]. When combined with low-dose dexamethasone, the most frequent nonhematologic AEs associated with pomalidomide include fatigue and asthenia (47–63%) and infections such as upper respiratory tract infections (29–31%) and pneumonia (19–34%) [4]. The overall risk of SPMs in patients with MM is low, and although additional risk with pomalidomide treatment appears to be minimal, some cases of acute myelogenous leukemia have been reported in patients receiving pomalidomide in non-MM studies [4,5,15,16,17]. Although routine screening for SPMs is not recommended by the International Myeloma Working Group, they instead suggest bone marrow examination with cytogenetic analyses at baseline and in the event of unexplained blood count abnormalities. Further, they advise that SPMs should be assessed on a case-by-case basis to determine the role of treatment on SPM development to prevent false inflation of reported SPM rates [15]. A systematic review and meta-analysis of randomized controlled trials of RRMM revealed that, of 10 treatment regimens analyzed (including mono-, doublet-, and triplet-regimens), pomalidomide–dexamethasone regimens had the highest rate of infections [18]. Pomalidomide-associated AEs leading to death have also been reported, most commonly pneumonia, sepsis, and anemia [19]. Different pomalidomide-based combination regimens have demonstrated differing incidences of common AEs in clinical studies (Table 2), and effective management is important not only for patient QOL but also for treatment adherence and therefore overall treatment efficacy (Table 3).

### 1.1. Real-World Pomalidomide Data

Real-world data are consistent with clinical studies regarding the most frequently observed hematologic and nonhematologic AEs [33,34]. A recent pharmacovigilance analysis of >55,000 pomalidomide-related AEs between 2013 and 2021 based on the Food and Drug Administration’s (FDA) Adverse Event Reporting System (FAERS) has provided further insights into real-world post-marketing safety of IMiD agents (Table 4) [19]. The study concluded that IMiD agents showed differences in safety profiles and emphasized the importance of awareness amongst clinicians. The risk of VTE is high in MM and is increased by the use of IMiDs [35,36] and further still when IMiDs are combined with dexamethasone or chemotherapy drugs [37,38,39]. When compared with thalidomide (14%) or lenalidomide (15%), pomalidomide had the lowest risk of VTE (7%), possibly due to routine use of thromboprophylaxis in the treatment regimen [19,40]. Four significant system organ class safety signals were identified for pomalidomide: “respiratory, thoracic, and mediastinal disorders” (occurring in 4.9% of patients); “skin and subcutaneous tissue disorders” (occurring in 4.5% of patients); “musculoskeletal and connective tissue disorders” (occurring in 4.2% of patients); and “metabolism and nutrition disorders” (occurring in 2.8% of patients). The most frequently reported preferred terms for pomalidomide-associated AEs were pneumonia (occurring in 3.6% of patients), fatigue (occurring in 3.2% of patients), and decreased white blood cell count (occurring in 1.9% of patients) [19]. Of note, given that it is the most frequently observed AE in patients with RRMM treated with pomalidomide, neutropenia occurred in 1% of patients. These data should be caveated as the FAERS database is a spontaneous reporting system that likely leads to underreporting and incomplete or inaccurate reporting, resulting in bias in the results. In other real-world studies of pomalidomide, rates of neutropenia are more in keeping with those seen in clinical trials [41,42]. 

### 1.2. AE Management Guidelines

A consensus statement by the European Myeloma Network (EMN) recommends that patients treated with pomalidomide are monitored for hematologic events, thromboembolic complications, skin rash, infections, peripheral neuropathic pain, diarrhea, secondary malignancies, allergic reactions, dizziness, and confusion [28]. Further, pomalidomide dose modifications are indicated for grade 3/4 neutropenia or thrombocytopenia and for grade 2/3 skin rash [28].

A panel of myeloma experts’ consensus statement also provided guidance for the management of individual AEs of interest, covering neutropenia, infection, VTE, and peripheral neuropathy. It included recommendations regarding dose modifications to manage neutropenia, thrombocytopenia, rash, peripheral sensory neuropathy, and constipation, among other AEs, that can be used to improve treatment tolerability and reduce side effects [24]. However, dose modifications may have implications for efficacy, with previous studies of IMiD agents having shown significantly longer PFS in patients who remained on treatment for ≥12 months without dose reductions compared with those who had reductions within 12 months [43]. As a result, clinicians are sometimes reluctant to modify doses for fear of compromising efficacy. It should be noted, however, that in an analysis of lenalidomide-treated patients with RRMM who had received 1–2 prior lines of therapy, patients remained responsive to daratumumab, pomalidomide, and dexamethasone (DPd) when AEs were managed through dose modifications, which may have also prolonged the time to onset of AEs and improved outcomes [44]. Clinicians report that it can be challenging to manage pomalidomide-associated AEs appropriately in a real-world setting, especially in patients with high disease burden, comorbidities, a history of cytopenias, and when pomalidomide is combined with daratumumab or isatuximab. In general, with a starting pomalidomide dose of 4 mg, the dose should first be reduced to 3 mg and the AE burden reassessed. However, there remains a need for guidance around management using a scenario-driven case study approach. Here we present five hypothetical case studies of patients experiencing pomalidomide-associated AEs, with expert opinion regarding their management.

## 2. Case Studies

### 2.1. Case Study 1: Patient with Aggressive Relapse on Lenalidomide Maintenance Subsequently Treated with DPd

#### 2.1.1. Patient Background and Disease Characteristics

Patient 1 is a 65-year-old Caucasian male diagnosed with MM in January 2018. At diagnosis, Patient 1 had high bone marrow involvement (bone marrow plasma cell percentage, 65%). He initially received bortezomib, lenalidomide, and dexamethasone (VRd) with autologous stem cell transplantation (ASCT) in June 2018, followed by lenalidomide maintenance therapy. He experienced an aggressive biochemical relapse in January 2023. Patient 1 has cardiac comorbidities; he was diagnosed with hypertension in 2009, which is managed with amlodipine, and in 2020, he presented with congestive heart failure (CHF), New York Heart Association (NYHA) Class II, managed with carvedilol, furosemide, and valsartan/sacubitril. Further, due to the high disease burden in the bone marrow, Patient 1 has experienced thrombocytopenia (platelet nadir 40 × 10^9^/L) and neutropenia (absolute neutrophil count [ANC] nadir 0.7 × 10^9^/L).

Given the aggressive nature of Patient 1’s relapse, the most important consideration for treatment selection was disease control. Given the case history, a bone marrow biopsy to ascertain whether the cytopenias were due to heavy myeloma burden, residual toxicity from prior therapy, or a secondary marrow process was imperative to establish the potential reversibility. For patients experiencing their first relapse after ASCT, preferred regimens include an anti-CD38 antibody with dexamethasone combined with either carfilzomib or pomalidomide. Furthermore, existing comorbidities can play a role in treatment selection. Since this patient did not have high-risk cytogenetics (negative for del(17p), t(4;14), and t(14;14)) and data that suggested that DPd provided clinical benefit in patients with time to first relapse >2 years after ASCT [45], Patient 1 began treatment with DPd in 28-day cycles. Minimum recommended blood levels for starting pomalidomide at the full dose of 4 mg are ANC ≥1 × 10^9^/L and platelets ≥75/10^9^/L if <50% of bone marrow nucleated cells are plasma cells or ≥30/10^9^/L if ≥50% of bone marrow nucleated cells are plasma cells [24]. Although Patient 1 had experienced nadir below both these thresholds prior to starting therapy, given his aggressive disease, it was decided that a full starting dose of 4 mg should be used. With careful monitoring and support, Patient 1 subsequently achieved a very good partial response (VGPR) on DPd with cytopenias resolving with disease response. After approximately 1 year, Patient 1 continued to be in VGPR but cytopenias recurred. Once alternative causes were excluded, the pomalidomide dose was reduced to 2 mg, cytopenias again resolved, and Patient 1 remained in VGPR.

#### 2.1.2. Pomalidomide-Associated AEs and Strategies for Management

Close, frequent hematologic monitoring, especially for neutropenia, was particularly important for Patient 1 given their pre-existing cytopenia and aggressive clinical biochemical progression. Monitoring is also recommended in all patients treated with pomalidomide regardless of other risk factors [46]. Neutropenia with pomalidomide is usually short-lived (as opposed to that associated with cytotoxic chemotherapy) [24], and dose modification and management strategies have been established [25]. Dose interruptions and adjustments are the primary strategy to manage AEs; however, these will depend on the severity of the AEs [25]. Management with granulocyte colony-stimulating factor (GCSF) and/or platelet transfusions may be used; given Patient 1’s ANC nadir of 0.7 × 10^9^/L, GCSF supportive care during the first 1–2 cycles may be of particular benefit until Patient 1 achieved disease control. Frequency can be adjusted based on laboratory values throughout the cycle, and GCSF treatment stopped once ANC is consistently >1.0 × 10^9^ [24]. The goal should be to maintain intensity of therapy using supportive measures as appropriate, and doses may be increased if cytopenia resolves following treatment as expected following disease response.

Considering Patient 1’s history of cytopenia, he should be assessed for hypogammaglobulinemia and recurrent infections. Regardless of risk, grade 3/4 infections, especially pneumonia, are very common not only due to pomalidomide but also due to daratumumab and the disease itself [34]. It is recommended that patients receiving such triplet regimens are given antimicrobial prophylaxis including an antiviral drug such as acyclovir or valacyclovir, as well as an agent to prevent *Pneumocystis carinii* pneumonia, such as sulfamethoxazole/trimethoprim or dapsone [29]. In the event of infection, treatment should be interrupted until resolution, after which it may be restarted.

### 2.2. Case Study 2: Patient with Relapse on Lenalidomide Maintenance Subsequently Treated with DPd and Now in Remission

#### 2.2.1. Patient Background and Disease Characteristics

Patient 2 is a 58-year-old Caucasian female diagnosed with MM in April 2016. As with Patient 1, Patient 2 was initially treated with VRd and received ASCT in December 2016 followed by lenalidomide maintenance therapy. Patient 2 relapsed in October 2020 and started receiving DPd soon after. Patient 2 responded well to DPd therapy and experienced only mild AEs including nausea, constipation, and daratumumab injection-site reactions during the first two 28-day cycles. Following the first two cycles of DPd, Patient 2 complained of chronic fatigue, mental fog, and peripheral neuropathy (predominantly in the lower extremities) attributed by her physician to pomalidomide. By the end of the 16-week daratumumab administration phase, a significant reduction in disease burden was noted, and by the end of March 2021 Patient 2 had achieved a stringent complete response (sCR). Patient 2 continued the DPd regimen, with daratumumab now administered every 4 weeks, cycles of daily 4 mg pomalidomide for 3 weeks followed by 1 week off, and weekly dexamethasone.

#### 2.2.2. Pomalidomide-Associated AEs and Strategies for Management including Dose Modification

Dose modifications are a frequent and effective method to manage a range of AEs, although achieving a balance between efficacy and AE management is paramount. Given that Patient 2’s disease was well controlled, dose modifications could be considered to address AEs while allowing the patient to remain on therapy.

**Chronic fatigue**: Patients should be screened for multiple fatigue symptoms that may vary according to treatment and stage of disease. A focused history and physical examination should be taken prior to initiation of therapy and throughout treatment. Central sleep apnea is a potential cause of chronic fatigue yet is grossly underreported and underdiagnosed [31]; therefore, physicians may wish to eliminate this as a potential cause of fatigue before initiating other management strategies. Initial management should focus on education, counseling, and pain management. Additional general strategies for fatigue management that should be employed throughout include encouraging exercise and good nutrition [30], energy conservation strategies (e.g., setting priorities/expectations, delegating or eliminating certain activities, pacing oneself, ensuring to take sufficient rest periods, and ensuring that high-energy activities occur at times of peak energy), self-monitoring of fatigue levels, and use of distraction techniques. Anemia should also be assessed up-front and treated throughout as necessary. Once these general strategies are exhausted, further nonpharmacologic interventions include cognitive behavioral therapy. If fatigue persists, pharmacologic interventions such as psychostimulants, treatment for pain, emotional distress, or optimization of treatment for sleep dysfunction, nutritional deficit/imbalance, and comorbidities may be considered as appropriate [30]. Any treatable contributing factors should be treated as appropriate [30].

**Mental fog/sluggishness**: Similar to fatigue, management of mental fogginess should initially focus on lifestyle-oriented options. Cognitive exercises have been shown to improve cognitive function, and stress reduction techniques such as meditation and mindfulness may also help. Depression and anxiety may both contribute to mental fogginess, and appropriate emotional support should be provided in these cases [47]. Counseling focused on improving quality of sleep should be provided and sleep apnea ruled out. Dosing of treatment regimen in the evening may also reduce symptoms so may be considered. Other potential factors that may be investigated are vitamin B12 deficiency side effects related to pain relief medications.

**Neuropathy**: Peripheral neuropathy due to pomalidomide should be managed in much the same way as for other IMiD agents. Regular assessment to allow prompt recognition and subsequent monitoring of the severity and progression of neuropathy are vital [27]. Risk factors should be minimized, such as correcting vitamin B12 deficiency and utilizing appropriate diabetes management as needed [27]. For mild cases, pain management through gentle massage, warm baths, and cold/heat packs can be used. Pain medication such as gabapentin and pregabalin, tricyclic antidepressants, serotonin and norepinephrine reuptake inhibitors, carbamazepine, and opioid-type analgesics may be used depending on severity of pain [27]. Furthermore, physical activity and physical therapy can have benefit. Pomalidomide dose reductions may be considered based on the severity. For grade 3 peripheral neuropathy, dose should be interrupted until severity resolves to grade ≤1, after which it may be resumed at a lower dose. Grade 4 peripheral neuropathy necessitates discontinuation [24,27].

### 2.3. Case Study 3: Dosing Recommendations in a Patient with Renal Insufficiency

#### 2.3.1. Patient Background and Disease Characteristics

Patient 3 is a 62-year-old African American male diagnosed with MM in February 2016. He initially received bortezomib, cyclophosphamide, and dexamethasone, then ASCT in July 2016, after which he received lenalidomide maintenance therapy. Patient 3 experienced his first relapse in May 2019, after which he received bortezomib, thalidomide, and dexamethasone [48,49]; however, he experienced his second relapse in August 2020. Since his second relapse, Patient 3 has been receiving isatuximab, pomalidomide, and dexamethasone and has experienced manageable AEs, including mild fatigue and constipation. Patient 3 suffers from renal insufficiency; his current estimated glomerular filtration rate is 35 mL/min/1.73 m^2^, corresponding to stage 3b chronic kidney disease (CKD; moderate to severe); however, this has fluctuated significantly.

#### 2.3.2. Considerations for Treatment Selection

In individuals such as Patient 3, the extent and severity of renal insufficiency should be evaluated prior to selecting their next treatment and regimens associated with lower nephrotoxicity such as dexamethasone or bortezomib-based regimens considered. In patients with severe renal insufficiency, lenalidomide-based regimens can be used with caution with dose adjustments based on creatine clearance [48]. Reversal of renal failure in MM is achievable in approximately half of patients and results in improvements in long-term survival [50]. Pomalidomide undergoes extensive hepatic metabolism by CYP1A2 and CYP3A4 [51], so clearance does not rely on renal excretion, and studies have shown that 4 mg of pomalidomide daily plus low-dose dexamethasone is a viable treatment option in patients with RRMM and moderate or severe renal impairment, including those receiving hemodialysis [52]. Pomalidomide and dexamethasone (Pd) has shown good rates of complete renal response in patients with MM with comparable efficacy in patients with moderate renal impairment [53], and the addition of isatuximab to Pd has been shown to improve PFS, ORR, and renal response rates without dose adjustments being required [54]. DPd may also be considered in this setting. In patients with severe MM requiring dialysis, the pomalidomide US label recommends reducing the dose to 3 mg to be taken after completion of hemodialysis on dialysis days [4], although data from a phase 2 study indicate an acceptable safety profile with 4 mg in patients with moderate or severe renal impairment, including those requiring hemodialysis [52]. A similar approach may be taken in patients with fluctuating renal function. It is important to note that the label dosing was established in patients on hemodialysis rather than peritoneal dialysis. Peritoneal dialysis can be highly patient dependent, and close collaboration with a nephrologist is essential in these cases.

#### 2.3.3. Pomalidomide-Associated AEs and Strategies for Management

Management of AEs in Patient 3 should be considered in the context of his renal insufficiency. Infections are recognized as an important complication among patients with CKD, as such management of infections in these patients is important [55]. Additionally, MM and its treatment increase susceptibility to infection [24]. Thus, antimicrobial prophylaxis is of paramount importance [56]. Ensuring patients and household members are up-to-date with recommended vaccinations is also advised.

**VTE:** Randomized studies regarding the best thromboprophylaxis are lacking, with many centers continuing to use aspirin in low-risk patients; nonetheless, all patients with MM treated with pomalidomide should be on thromboprophylaxis [4,57]. Appropriate VTE prophylaxis will depend on a patient’s individual risk factors, comorbidities, and severity of renal insufficiency. In patients at high risk of VTE, a prophylactic dose of low-molecular-weight heparin or an international normalized ratio 2–3 of vitamin K antagonist (e.g., warfarin) is advised [24], although aspirin is a frequently used option that is both convenient and effective [58]. Dose adjustments are generally required for anticoagulant medications. For example, a prophylactic rivaroxaban dose of 10 mg once daily is recommended in patients with creatinine clearance (CrCL) ≥15 mL/min, whereas dosing is not recommended in those with CrCl <15 mL/min [59]. The recommended prophylactic apixaban dose is 2.5 mg twice daily, while 5 mg twice daily is recommended for VTE treatment, reduced to 2.5 mg twice daily in patients with two or more of the following characteristics: (i) age ≥80 years, (ii) body weight ≤60 kg, or (iii) serum creatinine ≥1.5 mg/dL. Further, in patients on hemodialysis, apixaban doses of 2.5 mg twice a day result in comparable drug exposure to 5 mg twice a day in patients with preserved renal function; 5 mg twice a day results in supratherapeutic apixaban levels and should therefore be avoided [60].

**Nausea and vomiting:** Antiemetic agents may be used to manage treatment-related nausea and vomiting. The American Society of Clinical Oncology (ASCO) classifies pomalidomide as having minimal emetic risk (<10%) and do not recommend routine antiemetic prophylaxis [61].

### 2.4. Case Study 4: Patient with a History of Intolerance to Lenalidomide (Rash, Diarrhea, etc.)

#### 2.4.1. Key Patient Clinical Characteristics/History

Patient 4 is a 58-year-old Hispanic male diagnosed with MM 6 years ago. He initially received lenalidomide, bortezomib, and dexamethasone (RVd) and achieved a VGPR. Despite responding well to treatment, Patient 4 quickly developed an intolerance to lenalidomide characterized by severe rash and gastrointestinal symptoms. Patient 4’s rash presented as an erythemateous, pruritic skin eruption that covered a significant portion of his body, which manifested as raised, well-defined plaques. As the rash progressed, it became increasingly painful and started significantly impacting Patient 4’s QOL. After initial monitoring and efforts to manage Patient 4’s symptoms using conventional methods, his healthcare team took the decision to discontinue lenalidomide and to assign Patient 4 to a new treatment regimen.

Considering his encouraging response but severe intolerance to lenalidomide, his oncologist switched Patient 4 to carfilzomib/pomalidomide/dexamethasone (KPd). Rash and diarrhea are generally less severe with pomalidomide treatment compared with lenalidomide. Nonetheless, pomalidomide was started at a low dose with the intention to titrate doses up if tolerated. Patient 4 has now achieved a VGPR and has been able to titrate to a full dose.

#### 2.4.2. Pomalidomide-Associated AEs and Strategies for Management

**Rashes:** In general, rashes are manageable. Limited, localized rash can be treated with antihistamines or topical steroids [32]. Mild-to-moderate rashes will tend to resolve with antihistamines and corticosteroids, although a temporary reduction in pomalidomide dose may be considered [32]. When rash is extensive, a short course of low-dose prednisone can also be used [62]. Pomalidomide dose modifications, interruptions, or discontinuation should be considered for grade 2/3 rashes if antihistamines and corticosteroids have proved insufficient, and permanent discontinuation should be considered for grade 4 events [4,28,32]. Once lesions clear, as for lenalidomide [32], pomalidomide treatment may be reinitiated, with a potential switch from dexamethasone to intermittent steroids. With appropriate management, severe recurrent rashes are extremely rare.

**Diarrhea**: Bile acid malabsorption can be a cause of persistent diarrhea in myeloma patients taking IMiDs [32] and bile acid sequestrants may therefore be considered to manage pomalidomide-induced diarrhea.

### 2.5. Case Study 5: Patient with Other Side Effects (Including Financial and Socioeconomic Factors)

#### 2.5.1. Patient Background and Disease Characteristics

Patient 5 is a 62-year-old Caucasian female diagnosed with MM 8 years ago. After initial treatment with RVd, she achieved a partial response; however, she experienced multiple relapses over the years and her disease became refractory to standard therapies. Recently, her oncologist recommended a pomalidomide-based regimen to manage her RRMM and she was given KPd.

Patient 5 lives in a small rural town and has financial limitations, resulting in her often struggling to afford medications and travel expenses for her frequent medical appointments. To support her treatment, she enrolled in a patient assistance program to help cover the costs. Since starting KPd, Patient 5 has experienced some side effects, including fatigue, nausea, and neutropenia.

#### 2.5.2. Financial Toxicity

Financial issues can affect patients with cancer, including those with RRMM, in several ways. Patients may miss appointments or may discontinue treatment due to the high cost of medications or financial constraints, resulting in suboptimal treatment outcomes [63,64]. Patients with lower socioeconomic status may also have reduced health literacy due to limited access to healthcare resources and education. This can lead to decreased understanding of the disease, treatment options, and potential side effects, resulting in poor treatment adherence, mismanagement of side effects, and reduced QOL [64]. Difficulties affording transportation to healthcare facilities for treatment and follow-up appointments are also a concern, and missed work can exacerbate financial burden problems, leading to suboptimal access to care and delays in treatment. Pomalidomide-based regimens are relatively expensive, with access for some patients being dependent on prescription benefit coverage or pharmaceutical company patient assistance programs. This means that, due to financial considerations, many patients may not receive optimal treatment or may experience treatment delays. Several organizations including the American Society of Hematology (High-Cost Hematologic Drug Access) and Leukemia & Lymphoma Society (Susan Lang Pay-It-Forward Patient Travel Assistance Program [lls.org]) have developed supportive programs for patients with hematologic conditions who face significant financial needs. These resources can help clinicians and patients access high-cost hematologic drugs and cover the cost of treatment-related travel and lodging expenses.

## 3. Future Directions

Targeted protein degraders (TPDs), including novel cereblon E3 ligase modulator (CELMoD™) agents such as iberdomide (CC-220) and mezigdomide (CC-92480), are an emerging class of agents [65,66,67,68,69,70,71]. Like pomalidomide, both iberdomide and mezigdomide target cereblon to induce ubiquitination and subsequent degradation of Ikaros and Aiolos; however, novel CELMoD agents have demonstrated enhanced tumoricidal activity and greater immunostimulatory activity compared with classic IMiD agents in preclinical models [65,66,67]. CELMoD agents have also shown promising efficacy in early-phase clinical studies (Table 5) [71,72].

Safety data with novel CELMoD agents are also encouraging. In the dose expansion of a phase 1/2 study, iberdomide plus dexamethasone had a manageable safety profile in heavily pretreated late-line RRMM, with 82.2% of patients experiencing grade 3/4 treatment-emergent AEs (TEAEs). The most common hematological grade 3/4 AEs were neutropenia (44.9%, 4.7% febrile neutropenia), anemia (28.0%), thrombocytopenia (21.5%), and leukopenia (20.6%). Infections were the most common nonhematological AEs, with 27.1% of patients having grade 3/4 infections including 8.4% with grade 3/4 pneumonia [71]. Grade 3/4 diarrhea occurred in 1% of patients, rash in 3%, and fatigue in 4% [71].

In the dose-expansion cohort of another phase 1/2 study, mezigdomide plus dexamethasone showed a manageable safety profile. The most frequent grade 3/4 AEs were neutropenia (75%, with 15% febrile neutropenia), anemia (36%), and thrombocytopenia (28%). Grade 3/4 infections occurred in 35% of patients. Grade 3/4 diarrhea occurred in 3% of patients, constipation in 0%, and fatigue in 5%. Overall, AEs resulted in dose reductions in 25% of patients, and 6% discontinued due to AEs [72].

Cemsidomide (CFT7455) is a non-CELMoD next-generation TPD targeting Ikaros/Aiolos that is currently being investigated in a phase 1 clinical trial [73]. In preclinical models, cemsidomide demonstrated sustained IKZF1/3 degradation and durable tumor regressions for a prolonged period after drug discontinuation [68]. Neutropenia (80%), thrombocytopenia (60%), anemia (20%), and leukopenia (20%) were observed in early clinical data, although the sample size was very small and in very heavily pretreated patients (N = 5) [68].

Work to date on these CELMoD agents has demonstrated promising efficacy, meaning they provide exciting potential future treatment options. Data suggest that novel CELMoD agents are associated with similar types of hematologic AEs as classic IMiD agents but with lower grade 3/4 nonhematologic toxicities [2,3,4]. However, these will need to be monitored as more data become available to allow effective management strategies to be developed, especially with potential longer-term use, as has been commonly observed with drugs in this class [2,3,4]. Further, these longer-term data will inform efficacy and safety comparisons of TPDs with other novel therapies in development for RRMM.

## 4. Conclusions

As outlined in this article, different IMiD agents exhibit distinct safety profiles. While general recommendations regarding treatment selection and AE management can be made, it is crucial to treat each patient individually. When selecting an appropriate treatment, healthcare practitioners should consider multiple factors covering patient-, disease-, and treatment-related factors [74].

Healthcare practitioners should also be mindful of when it may be appropriate to reduce or discontinue treatment in response to AEs. This decision depends on several factors, including the type and severity of the AE and the patient’s response to treatment. For nonhematologic AEs, grade 1/2 events can generally be managed using standard interventions, whereas grade 3/4 events may require dose modifications or treatment discontinuation [24]. Hematologic adverse events such as neutropenia and thrombocytopenia, as well as VTE, are all of particular interest in patients with RRMM treated with pomalidomide and require special attention, monitoring, and management, often including dose modifications [4,24].

In addition to dose reduction and temporary discontinuation of pomalidomide therapy in response to AEs, lower starting doses and modified dosing schedules are potential strategies for reducing toxicity while sustaining efficacy [75]. Data from a phase 2 nonrandomized trial (NCT00558896) showed no efficacy advantage for 4 mg versus 2 mg dosing when combined with dexamethasone [75]. Furthermore, data may also suggest that pomalidomide doses lower than the currently approved standard of 4 mg may provide sufficient clinical efficacy while reducing the incidence and severity of adverse events [76]. Further clinical research may provide further insights into the effectiveness and safety of such approaches.

AEs are common in patients receiving pomalidomide; however, appropriate management can minimize their impact on dosing, patient health, and QOL. Thromboembolic events, peripheral neuropathy, and hematologic toxicities are all events of particular interest due to their frequency with pomalidomide treatment, but it is important to note that other AEs associated with pomalidomide use have not been addressed in this article [4]. Nonetheless, the underlying goals of selecting the appropriate regimens to minimize AEs, resolving AEs when they arise, and preventing further occurrences while maintaining treatment efficacy remain the same. Guidelines developed by expert panels from established organizations such as the International Myeloma Working Group and the National Comprehensive Cancer Network are valuable resources for healthcare practitioners and should be consulted [77]. Appropriate management, monitoring, and intervention strategies based on treatment guidelines, but adjusted on a case-by-case basis, could enable healthcare practitioners to optimize pomalidomide therapy for each patient.

With an increased number of treatments available, decision-making for patients with RRMM has become increasingly complex and should be based not only on efficacy but also on safety. Despite their very close structural and functional homology, pomalidomide and other IMiD agents have distinct safety profiles that in turn require unique management. Many common AEs associated with pomalidomide can be effectively managed in the real-world setting, maintaining QOL without reducing efficacy. The recent emergence of new therapies is expanding treatment options for patients with RRMM, but vigilance regarding AEs is required to optimize AE management, adherence to treatment, and in turn, long-term efficacy.

## Figures and Tables

**Table 1:** Any-grade adverse events occurring in ≥20% of patients in clinical trials of (A) thalidomide [2] ^a,b^, (B) lenalidomide [3] ^a,c^, and (C) pomalidomide [4] ^d^.

**A**		
**Body System**		**Thalidomide +** **Dexamethasone**
Adverse Event, %		
Hematologic		
Blood/bone marrow		
Decreased leukocytes		35
Decreased neutrophils		31
Nonhematologic		
General disorders and administration site conditions		
Asthenia		24
Gastrointestinal		
Constipation		50–55
Anorexia		28
Nausea		13–28
Musculoskeletal		
Muscle weakness		40
Metabolic/laboratory		
Hypocalcemia		72
Pulmonary		
Dyspnea		42
Dermatology/skin		
Rash/desquamation		30
Dry skin		21
Neurology		
Sensory neuropathy		54
Confusion		28
Anxiety/agitation		26
Tremor		26
Motor neuropathy		22
Dizziness/light-headedness		20–23
Constitutional symptoms		
Fatigue		21–79
Fever		24
Weight loss		23
Weight gain		22
Cardiovascular		
Edema		34–56
Thrombosis/embolism		22
**B**		
**Body system**	**Lenalidomide + dexamethasone**	**Lenalidomide +** **dexamethasone ^e^**
Adverse event		
Hematologic		
Anemia	44	36
Neutropenia	35	33
Thrombocytopenia	20	19
Nonhematologic		
General disorders and administration site conditions		
Fatigue	33	33
Asthenia	28	23
Pyrexia	21	19
Gastrointestinal disorders		
Diarrhea	45	39
Abdominal pain	20	14
Musculoskeletal and connective tissue disorders		
Back pain	32	27
Muscle spasms	20	19
Respiratory, thoracic, and mediastinal disorders		
Cough	23	17
Dyspnea	22	16
Metabolism and nutrition disorders		
Decreased appetite	23	21
Skin and subcutaneous tissue disorders		
Rash	26	28
Psychiatric disorders		
Insomnia	28	24
**C**		
**Body system**	**Pomalidomide**	**Pomalidomide + low-dose dexamethasone**
Adverse event		
Hematologic		
Neutropenia	53	49–51
Anemia	38	<5–42
Thrombocytopenia	26	23–30
Leukopenia	13	13–20
General disorders and administration site conditions		
Fatigue and asthenia	58	47–63
Peripheral edema	25	17
Pyrexia	23	27–32
Gastrointestinal disorders		
Nausea	36	15–24
Constipation	36	22–37
Diarrhea	35	22–36
Musculoskeletal and connective tissue disorders		
Back pain	35	20–32
Musculoskeletal chest pain	23	20
Muscle spasms	21	15–20
Respiratory, thoracic, and mediastinal disorders		
Dyspnea	36	25–45
Cough	17	20–22
Metabolism and nutrition disorders		
Decreased appetite	23	13–19
Hypercalcemia	21	4–12
Skin and subcutaneous tissue disorders		
Rash	21	8–16
Infections and infestations		
Upper respiratory tract infection	37	29–31
Pneumonia	28	19–34
Nervous system disorders		
Dizziness	22	12–18
Peripheral neuropathy	21	17–18

^a^ Newly diagnosed multiple myeloma; ^b^ NCT00033332; ^c^ FIRST (NCT00689936); ^d^ NCT00833833; ^e^ 18 cycles.

**Table 2:** Common any-grade adverse events reported with pomalidomide-based combinations in clinical trials.

Study Name NCT Number	Phase	Line	Regimen	Most Common Hematologic Adverse Events		Most Common Nonhematologic Adverse Events	
				Any-Grade	Grade 3/4	Any-Grade	Grade 3/4
OPTIMISMM NCT01734928 [6]	3	≥2	PVd	Neutropenia (47%)	Neutropenia (42%)	Periphery sensory neuropathy (48%)	Pneumonia (11%)
				Thrombocytopenia (37%)	Thrombocytopenia (27%)	Constipation (37%)	Hyperglycemia (9%)
				Anemia (28%)	Anemia (14%)	Fatigue (37%)	Periphery sensory neuropathy (8%)
						Peripheral edema (34%)	Fatigue (8%)
						Diarrhea (34%)	Diarrhea (7%)
APOLLO NCT03180736 [20]	3	≥2	DPd	Neutropenia (70%)	Neutropenia (68%)	Fatigue (26%)	Pneumonia (11%)
				Anemia (37%)	Thrombocytopenia (17%)	Upper respiratory tract infection (23%)	Lower respiratory tract infection (11%)
				Thrombocytopenia (32%)	Anemia (17%)	Asthenia (22%)	Fatigue (8%)
				Leukopenia (26%)	Leukopenia (17%)	Diarrhea (22%)	Asthenia (5%)
				Lymphopenia (15%)	Lymphopenia (12%)	Pyrexia (20%)	Hyperglycemia (5%)
ELOQUENT-3 NCT02654132 [21]	2	≥3	EPd	Anemia (25%)	Neutropenia (13%)	Constipation (22%)	Hyperglycemia (8%)
				Neutropenia (23%)	Anemia (10%)	Hyperglycemia (20%)	Pneumonia (5%)
				Thrombocytopenia (15%)	Thrombocytopenia (8%)	Diarrhea (18%)	Bone pain (3%)
				Lymphopenia (10%)	Lymphopenia (8%)	Nasopharyngitis (17%)	Dyspnea (3%)
						Respiratory tract infection (17%)	
ICARIA-MM NCT02990338 [22]	3	≥3	IsaPd	Neutropenia (66%)	Neutropenia (64%)	Infusion reaction (38%)	Pneumonia (25%)
				Thrombocytopenia (17%)	Thrombocytopenia (15%)	Upper respiratory tract infection (36%)	Dyspnea (5%)
				Anemia (6%)	Anemia (5%)	Pneumonia (34%)	Lower respiratory tract infection (5%)
						Diarrhea (32%)	Cataract (5%)
						Bronchitis (28%)	Urinary tract infection (5%)
MMRC NCT01665794 [23]	1/2	≥2	KPd	Anemia (39%)	Lymphopenia (17%)	Fatigue (59%)	Asthenia (3%)
				Thrombocytopenia (32%)	Neutropenia (17%)	Upper respiratory tract infection (58%)	Lung infection (24%)
				Lymphopenia (32%)	Anemia (12%)	Dyspnea (50%)	Other infections (9%)
				Neutropenia (26%)	Febrile neutropenia (12%)	Diarrhea (44%)	Dyspnea (8%)
				Febrile neutropenia (14%)	Thrombocytopenia (9%)	Lung infection (36%)	

DPd, daratumumab/pomalidomide/dexamethasone; EPd, elotuzumab/pomalidomide/dexamethasone; IsaPd, isatuximab/pomalidomide/dexamethasone; KPd, carfilzomib/pomalidomide/dexamethasone.

**Table 3:** Management strategies for adverse events reported with pomalidomide-based combinations.

Adverse Event	Management Strategy
***Hematologic***	
Neutropenia [24,25,26]	Febrile neutropenia with ANC <0.5 × 10^9^/L: withhold pomalidomide, add GCSF, and follow complete blood count weekly; when ANC ≥1 × 10^9^/L, resume pomalidomide at dose 1 mg lower than before When ANC <0.5 × 10^9^/L but without fever, maintain pomalidomide dose and support with GCSF; if low ANC persists beyond 1 cycle, follow the guidelines for febrile neutropenia
Thrombocytopenia [4]	When platelets <25 × 10^9^/L, withhold pomalidomide until platelets are ≥50 × 10^9^/L; follow complete blood count weekly then resume at dose 1 mg lower than the previous dose
Anemia	General management strategies and supportive treatment
***Nonhematologic***	
Periphery sensory neuropathy [24,27]	Regular assessment/monitoring. Minimize risk factors. Pain management for mild cases Pomalidomide dose interruption for grade 3 cases until resolution to grade ≤1 then resumption at a lower dose. Grade 4: discontinue pomalidomide
Infection [24,28,29]	Routine vaccination recommended. Pomalidomide dose interruption until resolution. Consider antimicrobial prophylaxis in all patients but especially those at high risk or those receiving triplet regimens
Fatigue [30,31]	Focused history/physical examination. Eliminate sleep apnea as a potential cause. Education, counseling, pain management, exercise, good nutrition, energy conservation strategies, and use of distraction techniques may be implemented. Cognitive behavioral therapy may also be used. Pharmacologic intervention may be considered for persistent cases where other methods have failed
Diarrhea [32]	Consider bile acid sequestrants

**Table 4:** Top 20 preferred terms for reported numbers of real-world adverse events associated with (A) thalidomide, (B) lenalidomide, and (C) pomalidomide [19].

A		B		C	
Preferred Term	N ^a^ (%)	Preferred Term	N ^a^ (%)	Preferred Term	N ^a^ (%)
Thalidomide	21,045 (100)	Lenalidomide	460,923 (100)	Pomalidomide	102,810 (100)
Peripheral neuropathy	544 (2.58)	Diarrhea	15,527 (3.37)	Pneumonia	3683 (3.58)
Pneumonia	362 (1.72)	Fatigue	13,794 (2.99)	Fatigue	3299 (3.21)
Unevaluable event	306 (1.45)	Pneumonia	10,916 (2.37)	White blood cell count decreased	1980 (1.93)
Adverse drug reaction	288 (1.37)	Rash	9150 (1.99)	Asthenia	1328 (1.29)
Constipation	276 (1.31)	White blood cell count decreased	6627 (1.44)	Peripheral neuropathy	1306 (1.27)
Anemia	177 (0.84)	Peripheral neuropathy	5857 (1.27)	Constipation	1176 (1.14)
Deep vein thrombosis	171 (0.81)	Thrombosis	5506 (1.19)	Full blood count decreased	1158 (1.13)
Thrombosis	167 (0.79)	Constipation	5444 (1.18)	Neutropenia	1058 (1.03)
Hypoesthesia	167 (0.79)	Full blood count decreased	5301 (1.15)	Platelet count decreased	971 (0.94)
Peripheral swelling	142 (0.67)	Platelet count decreased	5295 (1.15)	Laboratory test abnormal	908 (0.88)
Renal failure	141 (0.67)	Muscle spasms	4205 (0.91)	Nasopharyngitis	903 (0.88)
Pulmonary embolism	134 (0.64)	Peripheral swelling	3751 (0.81)	Peripheral swelling	887 (0.86)
Full blood count decreased	131 (0.62)	Adverse drug reaction	3533 (0.77)	Back pain	859 (0.84)
Drug intolerance	131 (0.62)	Neutropenia	3500 (0.76)	Adverse drug reaction	814 (0.79)
Sepsis	124 (0.59)	Unevaluable event	3486 (0.76)	Thrombosis	810 (0.79)
Paresthesia	119 (0.57)	Anemia	3333 (0.72)	Unevaluable event	764 (0.74)
Infection	117 (0.56)	Laboratory test abnormal	3327 (0.72)	Influenza	763 (0.74)
Platelet count decreased	112 (0.53)	Hemoglobin decreased	3027 (0.66)	Infection	763 (0.74)
Neutropenia	112 (0.53)	Deep vein thrombosis	2880 (0.62)	Muscle spasms	728 (0.71)
Hemoglobin decreased	111 (0.53)	Dehydration	2687 (0.58)	Anemia	708 (0.69)

^a^ Number of patients with adverse events. Adapted from [19].

**Table 5:** Efficacy and safety of CELMoD agents in multiple myeloma in phase 1/2 clinical trials [71,72].

	Mezigdomide + Dexamethasone N = 101	Iberdomide + Dexamethasone N = 107 ^a^
ORR, % (95% CI)	41	26 (18–36)
Median time to response, weeks (IQR)	–	4.2 (4.1–10.9)
Median duration of response, months (95% CI)	7.6 (5.4–9.5)	7.0 (4.5–11.3)
Median PFS, months (95% CI)	4.4 (3.0–5.5)	3.0 (2.8–3.7)
Median OS, months (95% CI)	–	10.7 (8.8–NE)
Grade 3/4 TRAEs, %	–	82
Grade 3/4 infections, %	35	27
Grade 3/4 pneumonia, %	16	8

NE, not estimable; OS, overall survival; ORR, overall response rate; PFS, progression-free survival; TRAE, treatment-related adverse event. ^a^ Dose-expansion cohort.
